# Water, water everywhere! Any to drink?

**DOI:** 10.1093/conphys/coaa071

**Published:** 2020-07-31

**Authors:** Gail Schwieterman

**Affiliations:** Virginia Institute of Marine Science, William & Mary, Gloucester Point, VA, USA

Most animals are made up of more than 50% water. This means that getting water and keeping the right amount in your body are paramount to staying healthy and functional. For species that live in the ocean, however, maintaining the right balance of water inside the body can actually be pretty tricky. The salt in seawater draws freshwater out of the body via a process called osmosis, presenting a real challenge to marine mammals that need that fresh water to stay hydrated! Marine mammals might be able to overcome this challenge because of their unique physiology that helps them maintain the water levels they need inside their bodies. But, the water has to come from somewhere.

Could drinking saltwater actually prevent dehydration?

According to a team of researchers in Australia studying dugongs, the answer is… maybe.

Dugongs are marine mammals that are found in warm, coastal waters from East Africa to Australia, including the Red Sea, Indian Ocean, and the Pacific Ocean. They look similar to manatees, in that they are large, grey and have a roly-poly appearance, but dugongs have a dolphin-like tail that is different to the paddle-like tail of manatees. Unlike their manatee cousins, however, dugongs never enter fresh water. This means dugongs are always struggling to get the right balance of salt and water in their bodies. This difference between manatees and dugongs intrigued Laetitia Smoll and her team, and they designed experiments to find out how these animals survive.

To study this phenomenon, the team tested the salt concentration of the dugongs’ urine and blood. The results indicated that dugongs were capable of creating super salty urine—saltier than the ocean. This suggests that dugongs are actually able to extract freshwater from the seawater they drink and then release the excess salt. Thus, these animals can actually stay hydrated by drinking saltwater!

However, not all of the dugongs tested in this study had such extremely salty urine. In fact, the average level of salt in the urine from all of the dugongs studied was the same as the level in the ocean. So how do these animals stay hydrated?

To investigate this further, Smoll and her team looked for other potential water sources for dugongs. Dugongs are herbivores; they eat submerged plants like seagrasses. Most plants that live underwater contain more water than their land-based relatives. So, this could be a clue. Dugongs may be getting enough water from the plants they are consuming for food, but of course, more research needs to be done on the specific diets and consumption rates of dugongs.

Another potential way that dugongs are staying hydrated is by producing water inside of their bodies. Referred to as “metabolic water”, this is water created inside an animal when food is oxidized.

So, while dugongs *can* stay hydrated by drinking seawater, they likely do not use this physiological mechanism as their primary source of freshwater.

Given that dugongs are currently vulnerable to extinction, more research on the world’s only herbivorous marine mammal will help us fully understand how these gentle giants survive so we can better protect them and conserve their habitats.

Illustration by Erin Walsh; Email: ewalsh.sci@gmail.com



## References

[ref1] Smoll LI, Beard LA, Lanyon JM (2020) Osmoregulation and electrolyte balance in a fully marine mammal, the dugong (*Dugong dugon*). J Comp Physiol B, 1 –10. 31894351 10.1007/s00360-019-01250-8

